# Comparison in Antioxidant Potential and Concentrations of Selected Bioactive Ingredients in Fruits of Lesser-Known Species

**DOI:** 10.3390/foods13182926

**Published:** 2024-09-15

**Authors:** Barbara Łata, Piotr Latocha, Radosław Łaźny, Anna Gutfeld

**Affiliations:** 1 Section of Basic Research in Horticulture, Department of Plant Protection, Institute of Horticultural Sciences, Warsaw University of Life Sciences—SGGW, Nowoursynowska 159, 02-776 Warsaw, Poland; barbara_lata@sggw.edu.pl (B.Ł.); radoslaw_lazny@sggw.edu.pl (R.Ł.); anna.gu@o2.pl (A.G.); 2 Department of Environmental Protection and Dendrology, Institute of Horticultural Sciences, Warsaw University of Life Sciences—SGGW, Nowoursynowska 159, 02-776 Warsaw, Poland

**Keywords:** Actinidiaceae, Caprifoliaceae, Cornaceae, Elaeagnaceae, Rhamnaceae, Rosaceae, ascorbate, phenolics, total antioxidant potential

## Abstract

Fruits with a high content of biologically active compounds are essential in preventing many diseases. Therefore, the interest in searching for and testing new plant sources for bioactive constituents remains strong. Although many publications on individual species exist, their results are difficult to compare directly due to varying methods and conditions of analysis. Only a few studies have investigated many different species in a single analysis. Therefore, we examined and compared 21 different genotypes, using various measurement methods for total phenolic content (TPC) (Folin–Ciocalteu, FBBB), total antioxidant capacity (ABTS, FRAP, DPPH), and the HPLC technique for the total ascorbate concentration in freshly harvested fruits. One-way ANOVA, Principal Component Analysis, and Pearson Correlation were used to analyse and compare the results. The tested samples showed significant differences in TPC, ascorbate content, and antioxidant capacity. The correlation between the content of bioactive compounds and antioxidant capacity depended on the analytical methods, with results obtained using the FRAP test being the most strongly correlated. Due to higher levels of polyphenols, ascorbate, and antioxidant potential, the most promising species for further evaluation appear to be *Chaenomeles* × *californica*, *Actinidia kolomikta*, *Mespilus germanica*, and ×*Sorboaronia fallax*.

## 1. Introduction

Fresh fruits are characterised by the presence of numerous compounds with various structures and positive effects on the human body. These effects are commonly referred to as health-promoting, and fruits with a high content of biologically active compounds (also known as ‘super fruits’) are considered valuable in the prevention strategy for many diseases, including those related to ageing [1]. The interest in searching for and testing new plant sources (species or varieties) for bioactive constituents remains strong. The interest in this topic may also be reflected in the number of review reports published in recent years, including those on *Actinidia* [2,3,4] *Lonicera* [5,6,7,8], *Chaenomeles* [9,10,11], *Mespilus* [12,13], *Crataegus* [14], *Elaeagnus* [15], *Ziziphus* [16], and *Cornus* [17], among others. Recently, not only fruits (peel, flesh, endocarp) but also leaves, twigs, flowers, and seeds have been analysed for their concentration of biologically active compounds as potential food additives [2,4,18,19].

The chemical composition of fruits is mainly genetically determined [3,4,16,20,21]. However, the cultivation system (sustainable, organic), climate, and weather conditions during the growing season also significantly impact the composition [20,22,23]. Additionally, factors such as the stage of development and tissue type [16,18,20] play a role. Sometimes, tested fruits come from plants grown under well-defined conditions (soil type, fertilisation, and plant protection), but more often, they are sourced from places of natural occurrence or botanical gardens. Scientists provide knowledge, while farmers begin to include lesser-known, little-used, or forgotten plants in their cultivation [12,24]. Thanks to the wild or semi-wild conditions from which these plants originate, their resistance to biotic or abiotic stresses is high, and the likelihood of growing without pesticides increases. Not having chemical protection is essential for the quality of the fruit or other raw materials obtained from these plants [23].

Many studies and reviews have already been written about lesser-known fruits. Most often, they concern single fruit species or entire genera. Less often, they cover many species in the same laboratories, using selected methods, where it is possible to directly compare the quality of many genotypes and identify the most valuable ones for further, more detailed testing. Many factors influence the content of biologically active compounds, and the details of measurement (extraction method, type of examined tissue—fresh, dry, or lyophilised plant material) and measurement techniques make it difficult to compare test results from different laboratories worldwide. In the quality evaluations of these lesser-known fruits, clones, biotypes, or species are often considered as a whole and less frequently as well-defined genotypes (e.g., cultivars), further complicating comparative analysis, which indicates the need for further research. Special attention is paid to the concentration of phenolic compounds and total antioxidant activity [2,3,4,14,16,19,20,21,22,23,24,25]. In the case of L-ascorbate, precise detection methods are not always used [26].

Our study analysed 21 different genotypes, some of which were examined for the first time, using various measurement methods for phenolics and total antioxidant capacity and the HPLC technique for the total ascorbate concentration in freshly harvested fruits. We hope that this broader research will expand knowledge and enable a direct comparison in the biological value of lesser-known and still underutilised fruits.

## 2. Materials and Methods

### 2.1. Plant Material and Its Preparation

The subject of the evaluation was fruits of 21 plant genotypes at the consumer (eating) maturity stage. Full plant names, sources of origin, and botanical families, as well as acronyms used, are included in Table 1. The fruits were collected at the full ripeness stage (typical for each genotype) from plants growing predominantly in two locations to eliminate variability due to environmental factors and focus more on genetic differences. After harvest, fruit was washed in distilled water, dried, immediately frozen in liquid nitrogen, and then transferred to a low-temperature freezer (−23 °C). Before analysis, the fruits were homogenised in liquid nitrogen using a mortar and pestle. The obtained powder was transferred to cryotubes and stored at −80 °C. All analyses were performed in four biological replicates.

### 2.2. Bioactive Compound Measurements

#### 2.2.1. Ascorbate Determination

The first step was to weigh powdered fruit tissue into ice-chilled Eppendorf tubes containing 0.1 molar HCL and polivinylpolipyrrolidone (PVPP). Preliminary analyses were carried out to estimate the various fruits’ ascorbate content to adjust the mass of the weighed tissue and to establish the concentration range of the stock and working L-ascorbic acid solutions to the type of fruit being tested (poor vs. reach source of ascorbate). The starting mass was 50 mg of powdered material. The next step was centrifugation under the following conditions: 20 min at 4 °C at 21,900× *g*. Total ascorbate concentration was measured after complete oxidation of L-ascorbic acid to dehydroascobate with ascorbate oxidase. Next, the dehydroascorbate was derivatised with o-phenenylediamine, and the compound was detected fluorometrically at 450 nm by excitation at 350 nm with a Waters 474 Scanning Fluorescence Detector (Waters Co., Milford, MA, USA) under isocratic conditions. Separation condition: eluent—800 millimolar K_2_HPO_4_ in 20% methanol (pH = 7.8), flow rate 1 mL/min, column: Symmetry C18 column (150 mm, 4.6 µm, 5 µm, Waters Co., Milford, MA, USA). The final concentrations were calculated using a standard curve (stock solution 2.5–5.0 mg/mL depending on fruit material and expressed in mg/100 g fresh weight (FW).

#### 2.2.2. Total Phenolic Content (TPC)

TPC in fruit samples was measured by two methods: the first was with the Folin–Ciocalteu reagent, TPC (FOLIN), and the second method, using Fast Blue BB (4-benzoylamino-2,5-dimethoxybenzenediazonium chloride hemi [zinc chloride] salt, TPC (FBBB) [27,28] using a two-step extraction of powdered fruit tissue with methanol in an ultrasonic bath. Obtained extracts were diluted with redistilled water in the range 1:4 to 1:8 (*v*/*v*) depending on the species being examined and taking into account the absorbance readings for the standard curve. Gallic acid (GA) was used to prepare calibration curves (stock solution = 1 mg GA/mL and working standard solutions in the range 0–150 GA µg/L).

TPC (FOLIN). Extracts or standard solutions of 50 µL were transferred to Eppendorf tubes, and after adding 430 µL of double-distilled water and 20 µL of Folin’s reagent, the contents were mixed vigorously. The reaction was terminated by adding Na_2_CO_3_ (20%, 20 µL) and 450 µL of double-distilled water. After mixing and 1-h of incubation, the contents were transferred to couvetts and the absorbance was read at 725 nm in a HITACHI UV-VIS spectrophotometer (U-2900) (purchased from Dynamica Sci. Ltd., Milton Keys, UK).

TPC (FBBB). In this method, extracts or standards (with a volume of 1 mL) were transferred to Eppendorf tubes, then FBBB (0.1 mL 0.1% FBBB) and NaOH (5% NaOH, 0.1 mL) were added. Before adding 5% NaOH, the samples were mixed for 30 s. After one hour of incubation, absorption of obtained solutions was measured at 420 nm. All chemicals were purchased from Sigma Chemical Co., St. Louis, MO, USA.

In both methods, results were calculated using a standard curve and expressed in mg/100 g fresh weight (FW) of GA equivalents.

In both methods, TPC was expressed as gallic acid equivalents (GAE) in mg/100 g FW.

#### 2.2.3. Trolox Equivalent Antioxidant Capacity (TEAC)

Three different tests, described in detail, were employed to evaluate TEAC fruit tissue extracts: FRAP [29], ABTS [30] and DPPH assays [31]. Below is a brief description of particular methods.

FRAP-assay. The FRAP reagent contained 10 mM TPTZ (4,6-tri(pyridyl)-S-triazine) in 40 mM HCl, 20 mM FeCl_3_ and 300 mM acetate buffer (pH3.6) in a ratio of 1:1:10. The FRAP solution was prepared freshly every day and thermostated (5 min) at 37 °C before adding the sample extract and after. The volumes of FRAP reagent and test sample were 3 mL and 0.1 mL, respectively. When FRAP is mixed with the tested tissue extract, an intense blue colour is produced, with an absorption maximum at a wavelength of 593 nm.

ABTS-assay. The ABTS (2,20-Azinobis-3-ethylbenzotiazoline-6-sulfonic acid) radical was generated through the interaction of 19.2 mg of ABTS dissolved in 5 mL of HPLC-grade water and 88 µL of potassium persulfate (37.8 mg K_2_S_2_O_8_/mL H_2_O) in the dark at room temperature through for 16 h. After that time, the ABTS-activated radical was diluted with methanol to an absorbance of 0.70 ± 0.02 at 734 nm. The final solution contained 3.9 mL of diltuted ABTS and 0.1 mL of tested sample or standard. Absorbance was recorded after 1 h of incubation at 734 nm.

DPPH-assay. The DPPH (2,20-diphenyl-1-picrylhydrazyl stock solution was prepared by mixing 24 mg of DPPH radical with 100 mL of methanol. This stock solution was stored at −20 °C, and before determination, its absorbance was adjusted at 0.7 ±0.02 at 515 nm. The final solution contained 3.9 mL diluted DPPH and 0.1 mL of examined sample or standard. Absorbance was recorded after 1 h of incubation at 734 nm.

All chemicals were purchased from Sigma Chemical Co., St. Louis, MO, USA. The results of FRAP, ABTS and DPPH were then calculated using a calibration curve and expressed as mmol TE/kg FW (TE—Trolox equivalents; stock solution was freshly prepared 50 mmol of TE).

#### 2.2.4. Statistical Analysis and Presentation of Data

The results were analysed using one-way analysis of variance (ANOVA) and the multivariate Principal Component Analysis (PCA) biplot method with Statistica version 13.0 software (TIBCO Software Inc., https://www.tibco.com/, Santa Clara, CA, USA). The arithmetical means and standard deviations (SD) of the experimental data were calculated. The significance of the differences between mean values was evaluated using Tukey’s HSD test at a 5% probability level. Pearson correlation was employed to assess relationships between factors.

## 3. Results and Discussion

Due to their structure and biological activity diversity, phenolic compounds and L-ascorbate are among the most important determinants of fruit quality. Many studies on fruits, including the so-called lesser-known fruits, focus on analysing L-ascorbate and phenolics as fruits’ most prevalent and potent antioxidants. These studies often examine phenolic subgroups, such as flavonoids or anthocyanins [3,4,16,32] and/or individual compounds [2,3,33].

### 3.1. Ascorbate and Phenolics

L-ascorbate (L-ascorbic acid, vitamin C) is an essential hydrophilic antioxidant important for both animals and plants, playing a significant role in the antioxidant defence system and stress tolerance in living tissue [34,35]. Ascorbate performs a multitude of roles, which can be described elsewhere. Since humans cannot synthesise ascorbic acid, the primary source of this vitamin is fruits and vegetables. The concentration of ascorbate in the source, its availability throughout the year, and the amount consumed all influence the body’s supply of this nutrient. From a nutritional perspective, the biodiversity of consumed foods is crucial for human health due to the interrelationships between various compounds; for example, the maintenance of the reduced form of ascorbate depends on the presence of another antioxidant, glutathione. Selecting specific species and varieties as phytocompound donors is more acceptable than using biotechnological tools to increase their supply.

The presented study covered 21 different sources of biologically active compounds, species, and selected varieties in terms of fruit ascorbate and phenolic compound contents and total antioxidant capacity. A significant variation in the fruits’ total ascorbate was observed (1102-fold variation), with concentrations ranging from trace amounts, 0.71 mg/100 g of Fresh Weight (FW) in MGS to high levels (782.2 mg/100 g FW in AKDr) (Table 2). The average ascorbate concentration for the tested fruits was 132.0 mg/100 g FW. Fruits with an ascorbate concentration higher than the average included all varieties of *Actinidia kolomikta* (AKDr, AKT, AKV), CHM, and ZJ. *Chaenomeles* × *californica* ‘Gold Calif’ (CHGC) can also be considered a good source of L-ascorbate (100.6 mg/100 g FW). Apart from MGS, deficient concentrations of L-ascorbate were found in the fruits of *Elaeagnus multiflora* and *E. umbellata*, regardless of the cultivar. No significant differences were found among the cultivars within the selected species, except for AKV, which had a significantly lower ascorbate content compared to the other two cultivars of this species.

Pranckietis et al. [36] examined four varieties of *A. kolomikta*. They found a slightly lower range of vitamin C content (242.7 to 467.0 mg/100 g FW), with the varieties differing significantly in concentration. However, they used a less accurate colourimetric method (with dichloroindophenol). In turn, *Actinidia polygama* in this study had a much lower concentration of L-ascorbate (about 6 to 15 times less, depending on the variety) compared to *A. kolomikta*.

The genus *Actinidia* contains 54 species, of which the most economically important are *A. chinensis* var. *deliciosa* and *A. chinensis* var. *chinensis* known as kiwifruit [37]. Recently, *Actinidia arguta* (kiwiberry) has received much attention [4]. There are fewer reports regarding other species and their L-ascorbate content, such as *Actinidia eriantha*, *A. purpurea*, *A. melanandra*, *A. kolomikta*, or *A. polygama* [38].

The health-promoting properties and chemical composition of *Chaenomeles* were recently reviewed by several authors [9,10,11]. Species such as *Ch. thibetica*, *Ch. sinensis*, *Ch. speciosa*, *Ch. cathayensis*, *Ch. japonica*, and its hybrids like *Ch. × superba*, *Ch. × clarkiana*, and *Ch. × californica* belong to the *Chaenomeles* genus [10]. The chemical composition, especially the content of phenolic compounds and triterpenes of different Chinese varieties, was recently detailed [18,20]. Although the high L-ascorbate content in *Chaenomeles* is mentioned [9], comparative works on these species and hybrids are scarce. The Japanese quince cultivars we tested contained approximately 100.6 to 133.7 mg of ascorbate per 100 g of FW, consistent with another study [39], which reported 111–113 mg of L-ascorbate per 100 g FW. Apart from *A. kolomikta*, this concentration was higher than the other tested species.

Cornelian cherry (*Cornus mas*) is another species considered a valuable source of phytocompounds and genetic stock for breeding programmes [23]. Cornelian cherry has garnered significant interest, with many scientific publications and review reports devoted to its properties [17,40,41]. L-ascorbate is a trait that may distinguish Cornelian Cherry from others. In this study, *C. mas* was represented by four cultivars with ascorbate concentrations ranging from approximately 22.1 to 51.7 mg/100 g FW (Table 2). The CMJ variety had the highest concentration, and the CMF variety had the lowest. The fruits of *Ziziphus jujuba* were also a rich source of ascorbate, with concentrations above 400 mg per 100 g FW. Slightly lower contents were determined by Chen et al. [42] when testing five varieties and by Taherogorabi et al. [43], with 192–359 mg/100 g. Similar ranges were reported by Skender et al. [23] for 22 genotypes grown in northwestern Bosnia and Herzegovina (16–59 mg/100 g), six cultivars grown in Poland [44] (34–75 mg/100 g), and fruits from Italy [45] (61 mg/100 g). However, the L-ascorbate content can reach higher values [46], and as analysed in the review by Szczepaniak et al. [40], the concentration range may also depend on the determination method. Higher concentrations, up to 300 mg, were obtained using spectroscopic or iodometric techniques [40]. Species that could also deserve attention for L-ascorbate content are CAZ, LC, and SAR. However, these species were represented by only one cultivar, so it is worth considering extending the analysis to a larger number of genetic resources. Ascorbate concentrations for CAZ, LC, and SAR were 33.0, 50.9, and 64.5 mg per 100 g FW, respectively (Table 2). A study on eighteen genotypes of *Crataegus* representing four species (*C. monogyna*, *C. atrosanguinea*, *C. orientalis* var. *orientalis*, and *C. meyeri*) grown in Turkey revealed mean fruit ascorbate levels ranging from 23 to 30 mg/100 g FW, depending on the species [47]. Some genotypes had concentrations below 20 mg and above 30 mg of ascorbate per 100 g FW. These results are similar to the concentrations obtained in this study for CAZ. In the case of the *Lonicera* species, a wide range of cultivars and clones from different countries have been researched, showing a variation in L-ascorbate concentration [22,24,32]. Eleven *Lonicera* cultivars from central Lithuania exhibited L-ascorbate content in the 15–54 mg range per 100 g FW [32]. Higher L-ascorbate contents were recorded for *Lonicera* cultivars and clones tested in Slovakia, with 100–142 and 68–187 mg per 100 g, respectively [24]. Bozhuyuk [48] tested 12 pre-selected genotypes of *Sorbus aucuparia* with larger fruit, free from pests and diseases, and recorded only a 1.35-fold variation in fruit L-ascorbate content (28.4–38.2 mg per 100 g FW). In the case of other species (all cultivars of *Elaeagnus*, MGS), the obtained concentrations of L-ascorbate ranged from 7 to 27 mg/100 g (Table 2). From a nutritional point of view, even a small amount of L-ascorbate a frequently consumed product is an important element of the diet. However, in the case of these fruits, there will probably be no such relationship. Research on four biotypes of goumi (*Elaeanus multiflora*) showed low L-ascorbate concentrations, ranging from approximately 4 to 8 mg per 100 g FW [49].

In the case of *Mespilus germanica*, Popovic-Djordjevic et al. [12] reviewed studies on the chemical composition of this species, including detailed L-ascorbate concentrations, providing the analytical method used. The concentration of L-ascorbate depended on the extraction method and generally did not exceed 20 mg per 100 g FW, with some studies reporting levels of 40–50 mg per 100 g FW [13]. In this study, the content of ascorbare was low and ranged from aprrox. 1 (MGS) to 11.1 mg per 100 g FW.

Research on phenolics often covers their total content and subgroups, such as flavonoids or anthocyanins [3,4,16,32] and/or individual compounds [2,3,33]. In our study, two methods were used to determine the total content of phenolic compounds: one with the Folin–Ciocalteu reagent and the other with the FBBB reagent. The first method has been used for a long time, while the second was developed in 2011 [27]. On average, TPC (FBBB) (150.5 mg/g FW) was approximately 34% higher than that determined by the Folin–Ciocalteu method (112.6 mg/100 g FW) (Table 2). However, this relationship was not always consistent. Notably, high deviations from this rule occurred in the tested varieties of *A. kolomikta*, irrespective of cultivar and CMF, with more minor deviations in AP, EM, MGS, and ZJ. Correlation analysis of the results obtained by both methods did not show a significant relationship (*r* = 0.23), confirming the different mechanisms of the two assessment methods.

A highly positive correlation (*r* = 0.73, *p* < 0.001) was found between the ascorbate concentration and TPC (FOLIN), whereas no significant correlation (*r* = −0.24) was observed between TPC (FBBB) and the ascorbate concentration (Table 3). In the case of very high L-ascorbate concentrations in *A. kolomikta* cultivars or ZJ, the TPC measured using the Folin–Ciocalteu reagent was from 2 (ZJ) to 10 (AKDr) times higher compared to the concentration measured using the FBBB reagent. As suggested by some authors [27], the TPC (FOLIN):TPC (FBBB) ratio may depend on the ratio of the content of reducing non-phenolic to phenolic compounds in a given source. At a high concentration of reducing non-phenolic compounds, this ratio may increase above 1. For example, *A. kolomikta* species, characterised by very high ascorbate content in this study, had a TPC (FOLIN):TPC(FBBB) ratio ranging from 4.5 (AKT) to 10.2 (AKDr). These differences may, but only partially, explain the discrepancy between these two methods of phenolic measurement. For instance, *Chaenomeles* × *californica* (CHGC, CHM), described as a species with a relatively high concentration of ascorbate among the tested ones, was characterised by an almost three-fold higher concentration of TPC (FBBB) compared to TPC (FOLIN). It can be expected that high TPC (FOLIN) values, as opposed to TPC (FBBB), are associated with high concentrations of other non-phenolic compounds. Evaluating both methods requires further, more extensive, and detailed research.

The average content of TPC (FOLIN) ranged from 6.6 mg/100 g FW (SP) to 309.6 mg/100 g FW (AKV), while TPC (FBBB) ranged from 20.6 mg/100 g FW (CMF) to 482.9 mg/100 g FW (MGA). Among the tested species and cultivars, there was a high variation of phenolic compounds (47-fold variation for TPC-FOLIN and 23-fold variation for TPC-FBBB). In both methods, high concentrations (i.e., above the average for a given method) of phenolic compounds were found in *Chaenomeles* × *californica* (both cultivars), LC, and MGA. The literature provides data on the phenolic compounds of other species, such as *Chaenomeles sinensis* and *Ch. speciosa* [18,20]. Additionally, testing of the peel, pulp, and endocarp revealed that the highest content of phenolic compounds was found in the endocarp [18]. Comparing the peel and flesh [20], the peel was a better source of total flavonoids and phenolics. Due to the specificity of the examined tissues, direct comparisons with the present studies are difficult. Byczkiewicz et al. [39] reported the total phenolic compounds content determined by the Folin–Ciocalteu method for the ‘Gold Calif’ and ‘Maksym’ cultivars as 17.1 and 18.1 mg GAE per gram of dry matter (DM), respectively. Phenolic compounds in thirty different genotypes of *Lonicera caerulea* var. *kamtschatica* were characterised in detail by Kucharska et al. [50]. They found that the dominant subgroup was anthocyanins, with a total amount estimated to range from 150 to 654 mg per 100 g FW, depending on the variety. The fruits of the ‘Atut’ cultivar were classified as having a low content, i.e., 182 mg per 100 g FW. This difference underscores the importance of selecting cultivars with a broad spectrum of beneficial features, including chemical composition. *Mespilus germanica* has also been extensively tested for phenolic compounds, with studies assessing the fruits of this species as an important source of phenolics [51,52,53]. Tessa et al. [51] reported a total phenolic compound concentration for *M. germanica* of 138.6 mg per 100 g FW, a value close to the 174.9 mg determined for MGA in our study.

Apart from the species mentioned, the highest concentrations of TPC (FOLIN) (i.e., above average—112.3 mg/100 g) were recorded for AKT (188.9 mg), ZJ (199.7 mg), AKDr (260.0 mg), and AKV (309.6 mg), with all values in parentheses in mg per 100 g FW. Similarly, for TPC (FBBB), where the average concentration across all species was 150.5 mg/100 g FW, genotypes with above-average contents included EUK, CMSz, CMJ, CAZ, SFT, and MGA, with concentrations of 122.2, 156.5, 180.1, 207.0, 325.7, and 482.9 mg per 100 g FW, respectively. Therefore, species or selected cultivars that exhibit above-average concentrations of phenolic compounds in one or both methods can be considered potentially interesting in terms of their phenolic content. However, a comprehensive chemical composition analysis is required for a thorough assessment.

### 3.2. Total Antioxidant Capacity (TEAC)

The measure of the activity of biologically active compounds in plant tissues is their ability to scavenge free radicals. Since the antioxidant capacity of fruits is determined by a mixture of different biologically active compounds [20] with different mechanisms of action, different methods have, therefore, been developed to measure antioxidant capacity, which take into account the specificity of the compound/s. The most commonly used radicals in antioxidant capacity tests are ABTS•+ and DPPH•. These tests are based on the ability of an antioxidant to react with a radical generated in the test system. The DPPH test covers the antioxidant activity of hydrophilic compounds, and the ABTS works in both aqueous and organic extracts. In turn, the ferric reducing antioxidant power (FRAP) assay measures the reduction of ferric iron (Fe^3+^) to ferrous iron (Fe^2+^) in the presence of antioxidants, which are reductants. These methods are widely used to test antioxidant activity in foods and biological systems [54].

In our research, the total antioxidant capacity (TEAC), expressed as mmol TE/kg FW, varied between 1.83 (MGS) and 94.6 (AKV), 1.61 (SP) and 65.2 (CHM), and 8.66 (APP) and 121.3 (CHGC) for FRAP, ABTS, and DPPH, respectively (Table 4). These results indicate 52-, 41-, and 14-fold variations between the tested fruit samples for FRAP, ABTS, and DPPH, respectively.

High antioxidant capacity, i.e., above the average calculated for the tested species, was recorded in all tests for *Chaenomeles* × *californica* (CHGC and CHM), CMsz, and MGA. Values above average in at least two tests were found for the fruits of AKDr, AKT, CMJ, CMKM, EUA, MGS, and ZJ. The total antioxidant activity/capacity (TEAC) results from various biologically active compounds in plant tissues—hydrophilic and lipophilic antioxidants, which are usually strongly correlated with TEAC [55]. In our research, significant correlations were found only for FRAP and ascorbate content (*r* = 0.64, *p* < 0.05), TPC (FOLIN) content (*r* = 0.96, *p* < 0.001), and between the two methods, DPPH and ABTS (*r* = 0.80, *p* < 0.001) (Table 3). The high positive correlation obtained between the ABTS and DPPH tests in measuring the antioxidant capacity of the tested fruit species indicates that the fruit extracts showed comparable activity in both tests, although their values differed due to the specificity of the method, e.g., absorbance measurements were performed at different wavelengths. The interrelationships between these tests may vary in terms of the correlation coefficient [54]. It may probably depend on a given study variable as well as the variety of chemical composition of the material being tested [56]. Xu et al. [11] analysed available studies on *Chaenomeles* genus. They found that many compounds with biological activity have been extracted from this genus, including terpenoids, phenolics, flavonoids, phenylpropanoids and their derivatives, benzoic acid derivatives, biphenyls, oxylipins, and alkaloids. The authors of this review concluded that plants of the *Chaenomeles* genus have significant medicinal and nutritional value. Similarly, the *Cornus* is highly rated due to the presence of biologically active substances. In this case, the antioxidant properties are attributed to the high concentration of anthocyanins [57] but also to other polyphenols, iridoids [17,58], and L-ascorbate [59]. The diverse biological properties of *M. germanica* are attributed to its rich chemical composition, including carotenoids, amino acids, organic acids, proteins, vitamins, fatty acids, and a high concentration of phenolic compounds, which contribute to its antioxidant activity [13].

In this study, with a few exceptions (CMKM, EUA, MGS), species with high TEAC were also characterised by above-average concentrations of ascorbate and phenolic compounds (in one or both measurement methods used). However, when analysing all the tested genotypes, a significant correlation was found only for some adopted assessment methods (Table 3). These exceptions confirm that testing varieties are justified due to substantial differences, a point commonly highlighted by researchers in this field.

### 3.3. Multivariate PCA Biplot Assessment

**Figure 1:**
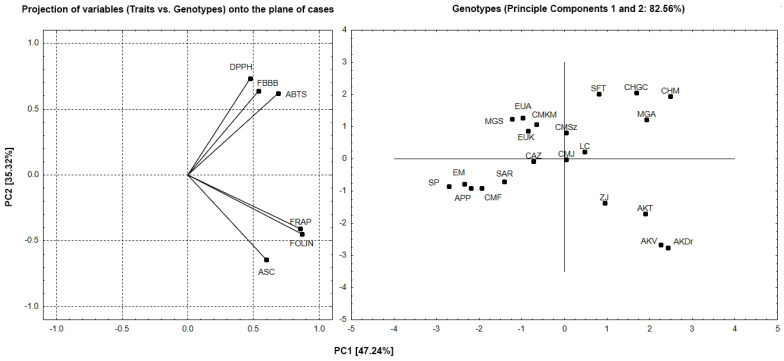
PCA biplot (PC1 vs. PC2) for 21 cultivars examined and their chemical and antioxidant properties.

To assess the interdependence of the tested genotypes and factors, a multivariate principal components analysis (PCA) was performed. The projection of variable factors responsible for 82.56% of the variability indicates the interdependence of DPPH, ABTS, and FBBB, as well as ASC, FOLIN, and FRAP (Figure 1). Both groups of factors are located in different but adjacent quadrants of the chart, suggesting a lack of a significant relationship between these groups.

A plot of the projection of cases onto the plane of factors allows us to group the studied genotypes based on their mutual similarity and differences and their relationship to the studied factors. As shown in Figure 1, the genotypes SFT, ChGC, CHM, and MGA were most positively correlated with TPC (FBBB), ABTS, and DPPH, while APP, CMF, SAR, EM, and SP were negatively correlated. Conversely, the ZJ, AKT, AKV, and AKT genotypes were positively associated with TPC (FOLIN), ASC, and FRAP, whereas the EUA, MGS, CMKM, and EUK genotypes were negatively correlated. The CMJ, LC, and CAZ genotypes showed no strong affinity for the tested factors. The analysis also confirms the differences in results depending on the evaluation method used.

## 4. Conclusions

Our research facilitated a direct and meaningful comparison of all tested genotypes regarding their biological value. Significant differences were observed in TPC, ascorbate content, and total antioxidant capacity. Additionally, some discrepancies were noted depending on the evaluation method used.

The variation in TPC and TEAC is related to the specificity of each determination method. For the total phenolic compound contents, the newer FBBB method was employed in addition to the commonly used Folin–Ciocalteu reagent method. Thus, as with antioxidant capacity, it is advisable to assess polyphenol content using multiple compatible methods. This approach provides a more comprehensive picture of the biological values of the tested materials.

Based on our research, the most promising species for further evaluation appear to be *Chaenomeles* × *californica*, *Actinidia kolomikta*, *Mespilus germanica*, and ×*Sorboaronia fallax*. Higher levels of phenolics, ascorbate, and antioxidant potential characterised all these genotypes. Further, more extensive research is needed to evaluate the differences between TPC analytical methods and analyse these fruits’ comprehensive chemical compositions.

Finally, it should be emphasised that, in addition to the genetic factor, variety is the main determinant of chemical compositions. Many external factors can be linked to variations in the quality of the fruits, and the species/variety should be tested in the region-specific soil and climatic conditions.

## Figures and Tables

**Table 1:** Description of genotypes investigated in the study.

Genotype	Common Name	Family	Fruit Origin	Cultivar Origin
*Actinidia kolomikta* ‘Dr Szymanowski’	Arctic kiwi	Actinidiaceae	Own collection	Poland
*Actinidia kolomikta* ‘Talin’	Arctic kiwi	Actinidiaceae	Own collection	Lithuania
*Actinidia kolomikta* ‘Vitakola’	Arctic kiwi	Actinidiaceae	Own collection	Czech Republic
*Actinidia polygama* ‘Pomarantseva’	Silvervine	Actinidiaceae	Own collection	Ukraine
*Chanomeles* × *californica* ‘Gold Calif’	Japanese quince	Rosaceae	Carya Nursery, Poland	Ukraine
*Chanomeles* × *californica* ‘Maksym’	Japanese quince	Rosaceae	Carya Nursery, Poland	Ukraine
*Cornus mas* ‘Szafer’	Edible dogwood	Cornaceae	Carya Nursery, Poland	Poland
*Cornus mas* ‘Flava’	Edible dogwood	Cornaceae	Carya Nursery, Poland	Unknown origin
*Cornus mas* ‘Jolico’	Edible dogwood	Cornaceae	Own collection	Austria
*Cornus mas* ‘Korałłowyj Marka’	Edible dogwood	Cornaceae	Carya Nursery, Poland	Ukraine
*Crategus* × *anomala* ‘Zbigniew’	Hawthorn	Rosaceae	Carya Nursery, Poland	Ukraine
*Elaeagnus multiflora* ‘Sweet Scarlet’	Cherry silverberry	Elaeagnaceae	Carya Nursery, Poland	Ukraine
*Elaeagnus umbellate* ‘K2’	Japanese silverberry	Elaeagnaceae	Carya Nursery, Poland	Poland
*Elaeagnus umbellata* ‘Amber’	Japanese silverberry	Elaeagnaceae	Carya Nursery, Poland	USA
*Lonicera caerulea* var. *kamtschatica* ‘Atut’	Honeysuckle	Caprifoliaceae	Carya Nursery, Poland	Poland
*Mespilus germanica* ‘Süssmispel’	Common medlar	Rosaceae	Carya Nursery, Poland	Germany
*Mespilus germanica* f. *apyrena*	Common medlar	Rosaceae	Carya Nursery, Poland	Natural origin
*Sorbopyrus auricularis* ‘Bulbiformis’	The shipova	Rosaceae	Carya Nursery, Poland	Czech Republic
*Sorbus aucuparia* ‘Rosina’	Rowan	Rosaceae	Carya Nursery, Poland	Germany
×*Sorbaronia fallax* ‘Titan’	Eesti	Rosaceae	Carya Nursery, Poland	Unknown origin
*Zizipus jujuba*	Jujube	Rhamnaceae	Kyiv Bot. Garden, Ukraine	Natural origin

**Table 2:** The total concentration of ascorbate and phenolic compounds depending on fruit species and cultivars tested (mg/100 g FW, average ±SD, *n* = 4).

Genotype	Acronym	ASC ^1^ ± SD	TPC (FOLIN) ^2^ ± SD	TPC (FBBB) ^3^ ± SD	FOLIN: FBBB Ratio
*Actinidia kolomikta* ‘Dr Szymanowski’	AKDr	**782.2** ± 327 ^c^	260.0 ± 24.0 ^n^	25.5 ± 1.4 ^a^	10.2
*Actinidia kolomikta* ‘Talin’	AKT	682.9 ± 1442 ^c^	188.9 ± 9.4 ^m^	42.0 ± 1.6 ^ab^	4.5
*Actinidia kolomikta* ‘Vitakola’	AKV	298.3 ± 592 ^b^	**309.6** ± 32.7 ^o^	51.6 ± 4.4 ^ab^	6.0
*Actinidia polygama* ‘Pomarantseva’	APP	51.4 ± 20 ^a^	50.0 ± 10.1 ^a–f^	30.8 ± 2.9 ^a^	1.6
*Chanomeles* × *californica* ‘Gold Calif’	CHGC	100.6 ± 16.8 ^a^	125.2 ± 23.5 ^i–k^	352.2 ± 28.9 ^h^	0.4
*Chanomeles* × *californica* ‘Maksym’	CHM	133.7 ± 21.0 ^a^	169.8 ± 21.2 ^k–m^	460.4 ± 35.4 ^i^	0.4
*Cornus mas* ‘Flava’	CMF	22.1 ± 2.5 ^a^	75.1 ± 16.2 ^c–h^	**20.6** ± 1.9 ^a^	3.7
*Cornus mas* ‘Jolico’	CMJ	51.7 ± 2.1 ^a^	101.5 ± 21.4 ^g–j^	180.1 ± 8.1 ^fg^	0.6
*Cornus mas* ‘Korałłowyj Marka’	CMKM	26.4 ± 9.8 ^a^	43.7 ± 4.9 ^a–e^	72.7 ± 4.6 ^bc^	0.6
*Cornus mas* ‘Szafer’	CMSz	29.7 ± 4.3 ^a^	90.5 ± 9.5 ^e–j^	156.4 ± 13.1 ^ef^	0.6
*Crategus* × *anomala* ‘Zbigniew’	CAZ	33.0 ± 6.0 ^a^	90.9 ± 13.1 ^f–j^	207.0 ± 14.5 ^g^	0.4
*Elaeagnus multiflora* ‘Sweet Scarlet’	EM	2.7 ± 0.13 ^a^	43.7 ± 6.8 ^a–d^	24.1 ± 5.6 ^a^	1.4
*Elaeagnus umbellata* ‘K 2’	EUK	1.3 ± 0.3 ^a^	58.9 ± 26.3 ^b–g^	122.2 ± 16.0 ^de^	0.5
*Elaeagnus umbellata* ‘Amber’	EUA	1.7 ± 0.4 ^a^	30.7 ± 9.2 ^a–c^	58.8 ± 4.0 ^a–c^	0.5
*Lonicera caerulea* var. *kamtschatica* ‘Atut’	LC	50.9 ± 2.8 ^a^	135.9 ± 12.8 ^j–l^	312.3 ± 6.0 ^h^	0.4
*Mespilus germanica* f. *apyrena*	MGA	11.1 ± 1.3 ^a^	174.9 ± 25.1 ^l–m^	**482.9** ± 29.5 ^i^	0.4
*Mespilus germanica* ‘Süssmispel’	MGS	**0.71** ± 0.1 ^a^	25.8 ± 3.3 ^ab^	26.9 ± 4.7 ^a^	1.0
*Sorbopyrus auricularis* ‘Bulbiformis’	SP	5.0 ± 0.1 ^a^	**6.6** ± 0.5 ^a^	29.6 ± 2.1 ^a^	0.2
*Sorbus aucuparia* ‘Rosina’	SAR	64.5 ± 1.1 ^a^	79.3 ± 23.0 ^d–i^	80.1 ± 12.1 ^bc^	1.0
×*Sorbaronia fallax* ‘Titan’	SFT	17.6 ± 4.3 ^a^	108.8 ± 3.7 ^h–j^	325.7 ± 31.7 ^h^	0.3
*Zizipus jujuba*	ZJ	403.7 ± 4.1 ^b^	199.7 ± 25.2 ^m^	98.9 ± 13.2 ^cd^	2.0
Average		132.0	112.3	150.5	
Fold variation between genotypes		1102	47	23	

^1^ the sum of L-ascorbic and dehydroascorbic acids; ^2^ expressed as gallic acid equivalent (GAE) and determined with the Folin–Ciocalteu reagent, TPC (FOLIN); ^3^ expressed as gallic acid equivalent (GAE) and determined using Fast Blue BB (4-benzoylamino-2,5-dimethoxybenzenediazonium chloride hemi [zinc chloride] salt, TPC (FBBB). Means followed by the same superscript letters in columns do not differ significantly; bolds refer to the highest and lowest values of the parameter given.

**Table 3:** Table of correlation coefficients between factors investigated.

	TPC (FOLIN)	TPC (FBBB)	ASC	FRAP	ABTS	DPPH
TPC (FOLIN)	X	0.23	0.73 ***	0.96 ***	0.27	0.06
TPC (FBBB)		X	−0.24	0.28	0.65 **	0.51 *
ASC			X	0.64 **	0.12	−0.07
FRAP				X	0.27	0.05
ABTS					X	0.80 ***
DPPH						X

Significance level: * *p* < 0.05, ** *p* < 0.01, *** *p* < 0.001.

**Table 4:** Total antioxidant capacity measured in ABTS (2,20-azino-bis(3-ethylbenzothiazoline-6-sulphonic acid), DPPH (2,2-diphenyl-1-picrylhydrazyl), and FRAP (ferric reducing antioxidant power) depending on fruit tested (mmol TE/kg FM; TE—trolox equivalents, average ± SD, *n* = 4.

Total Antioxidant Capacity, Assay Test		FRAP ± SD	ABTS ± SD	DPPH ± SD
Genotype	Acronym			
*Actinidia kolomikta* ‘Dr Szymanowski’	AKDr	68.2 ± 1.85 ^k^	30.9 ± 1.26 ^c–f^	53.0 ± 1.73 ^cde^
*Actinidia kolomikta* ‘Talin’	AKT	53.6 ± 0.54 ^j^	45.9 ± 10.4 ^f–h^	53.5 ± 13.6 ^de^
*Actinidia kolomikta* ‘Vitakola’	AKV	**94.6** ± 1.35 ^l^	20.8 ± 0.57 ^b–d^	35.8 ± 13.1 ^a–d^
*Actinidia polygama* ‘Pomarantseva’	APP	8.52 ± 0.29 ^b–d^	6.03 ± 0.47 ^ab^	8.66 ± 0.24 ^ab^
*Chanomeles* × *californica* ‘Gold Calif’	CHGC	34.6 ± 3.40 ^g^	63.8 ± 13.6 ^i^	**121.3** ± 13.5 ^hi^
*Chanomeles* × *californica* ‘Maksym’	CHM	46.3 ± 1.24 ^i^	**65.2** ± 2.07 ^i^	113.5 ± 13.7 ^g–i^
*Cornus mas* ‘Flava’	CMF	11.3 ± 0.04 ^cd^	10.2 ± 0.38 ^ab^	9.84 ± 0.08 ^ab^
*Cornus mas* ‘Jolico’	CMJ	41.9 ± 1.28 ^hi^	36.1 ± 0.48 ^e–g^	36.1 ± 2.10 ^b–d^
*Cornus mas* ‘Korałłowyj Marka’	CMKM	11.7 ± 1.88 ^cd^	51.4 ± 5.23 ^hi^	71.6 ± 21.6 ^ef^
*Cornus mas* ‘Szafer’	CMSz	31.1 ± 1.73 ^g^	36.5 ± 3.02 ^e–h^	93.3 ± 20.7 ^f–h^
*Crategus* × *anomala* ‘Zbigniew’	CAZ	22.8 ± 0.97 ^f^	27.4 ± 0.60 ^c–e^	17.6 ± 1.01 ^a–c^
*Elaeagnus multiflora* ‘Sweet Scarlet’	EM	9.89 ± 0.25 ^cd^	7.60 ± 0.59 ^ab^	6.00 ± 1.23 ^ab^
*Elaeagnus umbellata* ‘K 2’	EUK	12.9 ± 1.92 ^de^	30.8 ± 6.43 ^c–f^	85.2 ± 15.2 ^c–g^
*Elaeagnus umbellata* ‘Amber’	EUA	4.85 ± 0.95 ^ab^	44.7 ± 7.04 ^f–h^	90.6 ± 20.4 ^f–h^
*Lonicera caerulea* var. *kamtschatica* ‘Atut’	LC	39.6 ± 0.43 ^h^	37.0 ± 0.47 ^e–h^	29.9 ± 2.46 ^a–d^
*Mespilus germanica* f. *apyrena*	MGS	56.8 ± 4.04 ^j^	47.6 ± 2.64 ^gh^	74.0 ± 18.1 ^ef^
*Mespilus germanica* ‘Süssmispel’	MGA	**1.83** ± 0.35 ^a^	36.4 ± 5.28 ^e–h^	106.8 ± 21.0 ^f–i^
*Sorbopyrus auricularis* ‘Bulbiformis’	SP	8.26 ± 0.42 ^bc^	**1.61** ± 0.21 ^a^	**0.55** ± 0.18 ^a^
*Sorbus aucuparia* ‘Rosina’	SAR	17.4 ± 1.62 ^e^	16.8 ± 2.29 ^bc^	12.2 ± 1.73 ^ab^
*×Sorbaronia fallax* ‘Titan’	SFT	24.8 ± 3.47 ^f^	42.0 ± 8.93 ^e–g^	139.2 ± 10.6 ^i^
*Zizipus jujuba*	ZJ	39.4 ± 0.37 ^h^	34.7 ± 1.04 ^fg^	33.2 ± 1.65 ^a–d^
Average		30.5	33.0	56.8
Fold variation between genotypes		52	41	14

Means followed by the same superscript letters in each column do not differ significantly; bolds refer to the highest and lowest values of the parameter given.

## Data Availability

The original contributions presented in the study are included in the article, further inquiries can be directed to the corresponding author.
